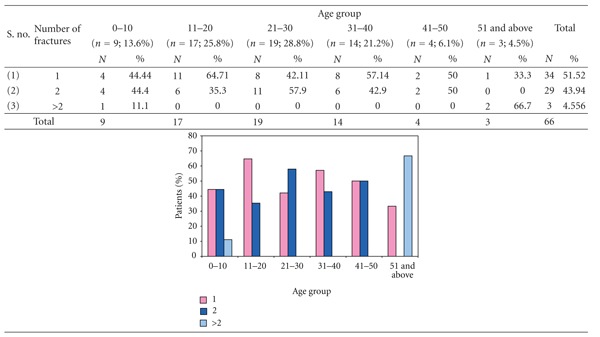# An Epidemiological Study on Pattern and Incidence of Mandibular Fractures

**DOI:** 10.1155/2012/834364

**Published:** 2012-11-08

**Authors:** Subodh S. Natu, Harsha Pradhan, Hemant Gupta, Sarwar Alam, Sumit Gupta, R. Pradhan, Shadab Mohammad, Munish Kohli, Vijai P. Sinha, Ravi Shankar, Anshita Agarwal

**Affiliations:** ^1^Department of Oral and Maxillofacial Surgery, Career Post Graduate Institute of Dental Sciences, Lucknow, India; ^2^Department of Oral and Maxillofacial Surgery, Babu Banarsi Das College of Dental Sciences, Lucknow, India; ^3^Department of Oral and Maxillofacial Surgery, Institute of Dental Sciences, Bareilly, India; ^4^Faculty of Dental Sciences, K.G.'s Medical College, Lucknow, India; ^5^Department of Oral and Maxillofacial Surgery, K.G.'s Medical College, Lucknow, India; ^6^Department of Oral and Maxillofacial Surgery, Saraswati Dental College, Lucknow, India; ^7^Department of Oral and Maxillofacial Surgery, Chandra Dental College, Lucknow, India; ^8^Department of Oral and Maxillofacial Pathology, Vananchal Dental College and Hospital, Garhwa, India

## Abstract

Mandible is the second most common facial fracture. There has been a significant increase in the number of cases in recent years with the advent of fast moving automobiles. Mandibular fractures constitute a substantial proportion of maxillofacial trauma cases in Lucknow. This study was undertaken to study mandibular fractures clinicoradiologically with an aim to calculate incidence and study pattern and the commonest site of fractures in population in and around Lucknow. Patient presenting with history of trauma at various centers of maxillofacial surgery in and around Lucknow were included in this study. Detailed case history was recorded followed by thorough clinical examination, and radiological interpretation was done for establishing the diagnosis and the data obtained was analyzed statistically. Out of 66 patients with mandibular fractures, highest percentage was found in 21–30 years of age with male predominance. Road traffic accidents were the most common cause of fracture with parasymphysis being commonest site. Commonest combination was parasymphysis with subcondyle. There was no gender bias in etiology with number of fracture sites. The incidence and causes of mandibular fracture reflect trauma patterns within the community and can provide a guide to the design of programs geared toward prevention and treatment.

## 1. Introduction

The sheer pace of modern life with high-speed travel as well as an increasingly violent and intolerant society has made facial trauma a form of social disease from which no one is immune. There are changes in patterns of facial injuries, extent, clinical features, and so forth resulting in mild-to-massive disfigurement of maxillofacial skeleton along with functional loss. 

Besides road traffic accident and violence, direct/indirect trauma may also occur due to sport activities, falls, and firearms. Occasionally, it may also be secondary to certain disease entities like cystic lesion, neoplasms, and metabolic diseases. 

The fracture is defined as “breach in the continuity of bone” [1]. Facial area is one of the most frequently injured area of the body, accounting for 23–97% of all facial fractures [2]. Mandible is the only mobile bone of facial skeleton and their has been a significant increase in number of cases in recent years. It is embryologically a membrane bone and is more commonly fractured than the other bones of face. Mandibular fractures occur twice as often as midfacial fractures [3]. The energy required to fracture it being of the order of 44.6–74.4 kg/m, which is about the same as the zygoma and about half that for the frontal bone [4–7]. It is four times as much force is required to fracture maxilla [8].

Bone fractures at site of tensile strain, since their resistance to compressive forces is greater [5]. Areas that exhibit weakness include the area lateral to the mental protuberance, mental foramen, mandibular angle, and the condylar neck [3]. The thickening on the inner aspect of the condylar neck or crest of the neck apparently acts as a main buttress of the mandible as it transmits pressure to the TMJ and the base of the skull.

The main causes of maxillofacial fractures worldwide are traffic accidents, assaults, fall, and sport-related injuries. Alcohol consumption is a well-known contributing factor to mandibular fractures derived from assault.

Hagan and Huelke in their survey showed a clean-cut pattern of mandibular fractures [9] as follows.

1. The Condyle region is the most common site of fracture.
2. Angle is the second most common site of fracture.
3. But if only one fracture is there, then angle is the most common site of fracture than condyle.
4. Multiple fractures are more common than single (ratio, 2 : 1), 4.80% of the patients were dentate.

Clinical examination may be sufficient to make a provisional diagnosis of a fracture, but the presence of edema, usually prevents an accurate assessment of the underlying skeletal damage. With maxillofacial radiography, at least two radiographs at right angles to each other are recommended. Because indirect fractures of the mandible are common, it is important to take radiographs at both sides of the jaw in every trauma case.

This study was undertaken to study various aspects of mandibular fractures clinically and radiologically with an aim to:

1. calculate the incidence of mandibular fractures;
2. study the pattern of fracture and the commonest site of fractures, in population in and around Lucknow.

## 2. Material and Method 

Patients presenting with history of trauma at various centers of maxillofacial surgery in Lucknow were included in this study.

Detailed information consisting of age, sex, socioeconomic status, chief complaint, history of present illness, past medical history, duration of injury, etiology, and associated injuries was recorded. After recording the history, a thorough clinical examination as well as radiological interpretation was done for each patient in this study for establishing the diagnosis. 

 Patients with history of trauma irrespective of clinical diagnosis of fracture were subjected to radiological examination to determine the diagnosis and to correlate with clinical examination findings to arrive at a diagnosis.

The data was analyzed in relation to age, sex, etiology of the fracture, site of fracture line, unilateral or bilateral, isolated fractures versus mandibular fractures with associated injuries, commonest combination of fracture site in mandible, interrelation of incidence of etiology and location of fracture; type of fracture whether single, double, or multiple with etiology, gender, and age, respectively.

 The statistical analysis was done using SPSS (Statistical Package for Social Sciences) Version 15.0 Statistical Analysis Software. The values were represented in frequencies and percentages. 

The following statistical formulas were used:

 (1) Chi square test:
  (1) $$\begin{array}{c} \chi^{2}=\frac{\Sigma {\left(O-E\right)}^{2}}{E}, \end{array}$$
  where *O* is observed frequency and *E* is expected frequency and
  (2)  level of significance: “*P*” is level of significance
   
*P* > 0.05 is not significant,
   
*P* < 0.05 is significant,
   
*P* < 0.01 is highly significant, and
   
*P* < 0.001 is very highly significant.

## 3. Results

### 3.1.  Table 1: Agewise Distribution of Study Subjects

Out of 66 patients, 37 had a unilateral mandibular fracture while 29 had bilateral fractures with maximum number of subjects were in the age group 21–30 years (28.8%) followed by 11–20 (25.8%), 31–40 (21.2%), <10 (13.6%), 41–50 (6.1%), and 60 years and above (4.5%). Around three-fourth (75.76%) of patients were in the age range 11 to 40 years.

### 3.2.  Table 2: Sexwise Distribution of Study Subjects

More than four-fifth (81.8%) of patients were males. Only 12 (18.2%) patients were female. The male to female ratio of the patients was 4.5 : 1. 

### 3.3.  Table 3: Etiologywise Distribution of Study Subjects

Road traffic accident (68.2%) was the cause of mandibular fractures in majority of subjects, followed by fall from height (30.3%) and hit against object (1.5%). 

### 3.4.  Table 4: Incidence of Mandibular Fractures According to Unilaterality/Bilaterality

56.1% patients had a unilateral mandibular fracture while 43.9% patients had bilateral fractures.

### 3.5.  Table 5: Mandibular Fractures and Associated Injuries

In 37.9% of cases, the mandible fracture was associated with other injuries while in majority (62.1%) no such associated injury was observed. 

### 3.6.  Table 6: Combinations

Among cases having multiple injuries (*n* = 32), fracture parasymphysis + subcondyle was the commonest (18.8%) followed by fracture body + angle (15.6%), fracture body + subcondyle (12.5%), and fracture parasymphysis + angle (12.5%). 

### 3.7.  Table 7: Site of Mandibular Fractures

Fracture parasymphysis (31.4%), body (24.5%), subcondyle (20.6%), and angle (13.7%) were the most common sites while fracture condyle (1%), coronoid (1.0%), dentoalveolar (1.0%), and ramus (1.0%) were the least common fracture sites. 

### 3.8.  Table 8: Association of Site of Mandibular Fractures with Etiology

Fracture parasymphysis was the most common fracture irrespective of the etiology. It was observed to be 30.6% of fractures with etiology fall from height and 31.8% of fractures with etiology road traffic accident. Fracture body was seen in 27.8% of fall from height and 22.7% of road traffic accident fractures while fracture subcondyle was seen in 22.2% and 19.7% fractures of fall from height and road traffic accident, respectively. Statistically, there was no significant difference in the site of fracture and type of etiology (*P* > 0.05). 

### 3.9.  Table 9: Age Group versus Number of Fracture Sites in Mandible

The patients in lower age group (0–10 years) and higher age groups (51 and above years) only had greater than two fracture sites. The number of patients with two fracture sites was maximum in the age group 21–30 years while it was proportionately lower in age group 11–20 and 31–40 years. 

## 4. Discussion

The sheer pace of modern life with high-speed travel as well as an increasingly violent and intolerant society has made facial trauma a form of social disease from which no one is immune. Seemingly, divergent shifts in society may be responsible for recent changes in patterns of facial injuries, extent, clinical features, and so forth resulting in massive disfigurement of maxillofacial skeleton. Mandible is the only mobile bone of facial skeleton, and there has been significant increase in the number of cases in recent years. Mandible fractures if not identified or inappropriately treated may lead to severe consequences both cosmetic and functional. 

This study was undertaken with the view to review the incidence, commonest site, and combination of mandibular fracture sites; to study corelation of site of fracture with etiology; to study correlation of number of fracture sites in mandible with age, sex, and etiology.

The incidence of mandibular fracture in this study increased with increasing age from 0 to 30 years then progressively decreased from 31 years of age. This could be explained as children till the age of 6 years are under parental care thereby prevented from sustaining severe injuries and the elasticity of bones makes them less prone to fracture. As the age progresses, they are more involved in physical activities, by the time they reach adulthood they are involved in fast and rash driving, interpersonal violence, alcohol abuse, contact sports, and so forth, while the people beyond 40 years of age lead a more calm, peaceful, and disciplined life.

In this study, the incidence was highest in 21 to 30 years of age (28.8%) followed by 11 to 20 years of age (25.8%); least being in 60 years and above (4.5%). This is in conformity with Adi et al. [10], Bataineh [11], Dongas and Hall [12], Ahmed et al. [13], Brasileiro and Passeri [14], but contradictory to Shapiro et al. [15] who reported 34.1 yearsas mean age range, Ogundare et al. [16]. 

Male are predominating with 81.8% while female constitute a meager percentage of 18.2%, that is, in a ratio of 4.5 : 1. This is in conformity with Adi et al. [10], Bataineh [11], Dongas and Hall [12], Ahmed et al. [13], Shapiro et al. [15], Ogundare et al. [16], Sakr et al. [17], and Brasileiro and Passeri [14] with a slight variation from this study. This is probably due to higher level of physical activity among men as they are still the bread winners in this part of the country.

Table 3 shows the etiologic division of study subjects. The most common etiologic factor in this study is road traffic accident (68.2%) which is in accordance with Luce et al. [7], Bataineh [11], Shah et al. [18], Ahmed et al. [13], and Brasileiro and Passeri [14]. Adi et al. [10], Dongas and Hall [12], and Olasoji et al. [19] reported assault as the main cause whereas no such case is reported in this study. In this study, fall from height is the second common etiologic factor accounting for 30.3% of the cases. Road traffic accident is still the major cause probably due to reckless and high-speed driving, reluctance to use helmets and seat belts, with inadequate enforcement of traffic safety rules. 

 In this study, out of 66 subjects 37 (56.1%) were reported as unilateral while bilateral accounted for 29 cases (43.94%), 62.12% were isolated mandibular fractures and 37.88% of cases had other associated injuries as mid-face fractures. This varied from the observations of Sakr et al. [17] who reported 91% cases as isolated mandible fractures and 9% cases with associated injuries. 

 Among the 102 fracture sites recorded in this study, the commonest site is the parasymphysis which accounted for a total of 32 followed by body (25), subcondyle (21), angle (14), symphysis (4), comminuted (2), ramus (1), condyle (1), coronoid (1), and dentoalveolar (1). The parasymphysis being the commonest in this study is contrary to Ellis et al. [20], Adi et al. [10], Bataineh [11], and Shah et al. [18] who reported body as the commonest while Dongas and Hall [12], Ogundare et al. [16], and Sakr et al. [17] reported angle; Motamedi [21], Ahmed et al. [13], and Brasileiro and Passeri [14] stated condyle as the most commonest site of fracture. 

The parasymphysis is probably the commonest site due to the presence of permanent tooth buds in the pediatric mandible presenting a high tooth to bone ratio, while in adults it is partly to the length of canine root weakening the structure.

The other reason for being the commonest site of fracture is as follows. The bone fracture at site of tensile strain since their resistance compressive force is greater. Mandible being similar to an architectural arch distributes the applied force along its length but not being a smooth curve in a uniform cross-section. There are parts at which force per unit area developed is greater resulting in increased concentration of tensile strength leading to a fracture at the site of maximum convexity of the curvature.

 The commonest combination of fracture in this study is parasymphysis with subcondyle accounting for 18.8%, probably due to the horizontally directed impact to parasymphysis resulting fracture at the site of impact, this axial force of impact against parasymphysis proceeded along the mandibular body to the cranial base through the condyle leading to the concentration of the tensile strain at the condylar neck hence resulting in its fracture.

 This is in contrary to Dongas and Hall [12] who found parasymphysis with angle, Ogundare et al. [16] reported body with angle as the commonest combination.

 The association of site of mandibular fracture with etiology had no significant variation, as the most common fractured site is parasymphysis followed by body and condyle showing the relation of site of fracture with point and intensity of impact rather that the etiological factor.

 The patients in lower age group, that is, 0–10 years and the higher age group, that is, 51 and above had greater than two fracture sites attributing to the higher tooth-to-bone ratio, thereby decreasing the bone mass among the lower age group and increased fragility of bone in higher age group.

## 5. Conclusions

The following conclusions have been drawn from the foregoing study.

The mandibular fractures were more common in males (81.8%) than females (18.2%) with the highest percentage in 21–30 years of age (28.8%), followed by 11–20 years of age (25.8%). Road traffic accidents were the most common cause of fracture followed by fall from height. 56.1% fractures were unilateral fractures and 62.1% were isolated fractures of mandible of which parasymphysis (31.4%) was the most common site of fracture in mandible followed by body (24.5%). There was only 1 case of coronoid fracture.

Commonest combination was parasymphysis with subcondyle followed by body and angle. There was no gender bias in etiology with number of fracture sites as the site of impact, intensity of trauma, and direction of force determine the number and fracture sites. Due to smaller sample size among various groups, statistical correlation was not possible; but patients in lower age group (0–10 years) and higher age group (51 and above) were more susceptible to multiple fracture sites.

## Figures and Tables

**Table 1:** Agewise distribution of study subjects (*n* = 66).

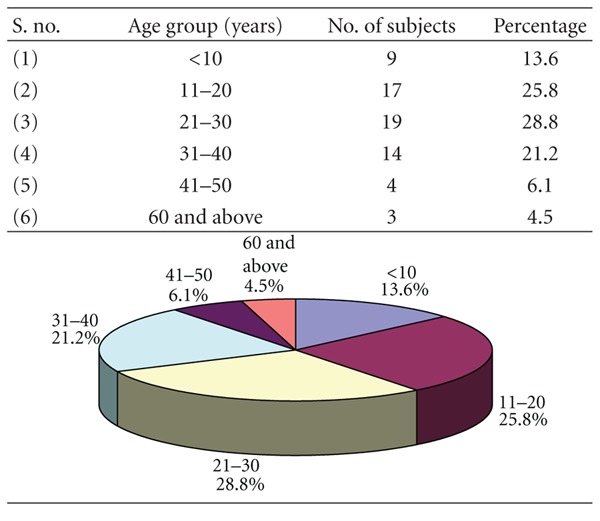

**Table 2:** Sexwise distribution of study subjects (*n* = 66).

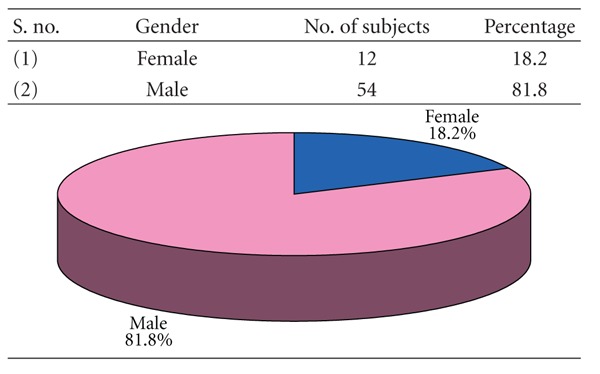

**Table 3:** Etiologywise distribution of study subjects (*n* = 66).

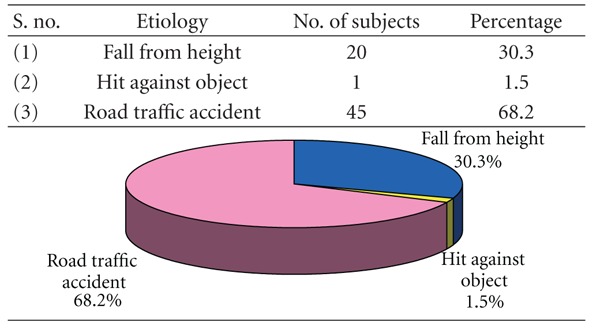

**Table 4:** Incidence of mandibular fractures according to unilaterality/bilaterality (*n* = 66).

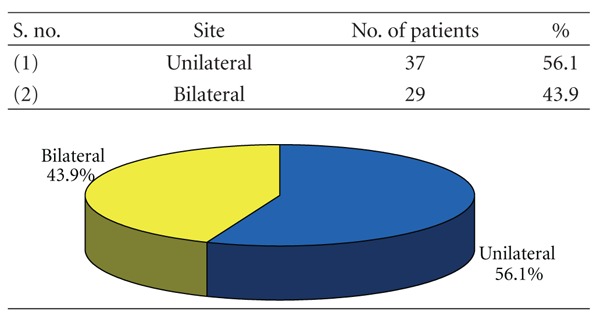

**Table 5:** Mandibular fractures and associated injuries (*n* = 66).

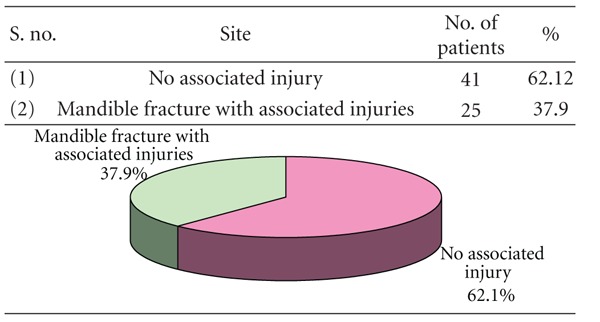

**Table 6:** Combinations (*n* = 32).

S. no.	Site	Number	% Age
(1)	Symphysis + subcondyle	2	6.3
(2)	Parasymphysis + body	3	9.4
(3)	Parasymphysis + angle	4	12.5
(4)	Parasymphysis + subcondyle	6	18.8
(5)	Parasymphysis + condyle	1	3.1
(6)	Parasymphysis + parasymphysis	2	6.3
(7)	Body + angle	5	15.6
(8)	Body + subcondyle	4	12.5
(9)	Body + body	2	6.3
(10)	Subcondyle + subcondyle	1	3.1
(11)	Ramus + parasymphysis	1	3.1
(12)	Dentoalveolar + subcondyle	1	3.1

**Table 7:** Site of mandibular fractures.

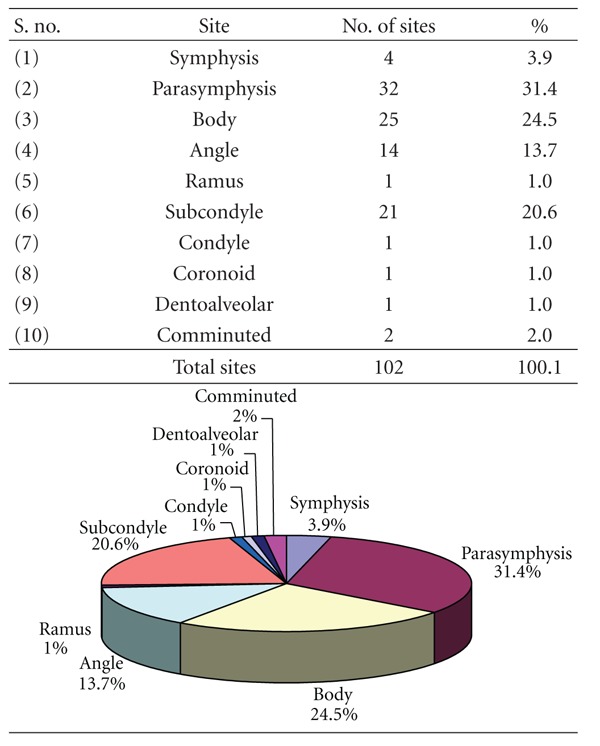

**Table 8:** Association of site of mandibular fractures with etiology.

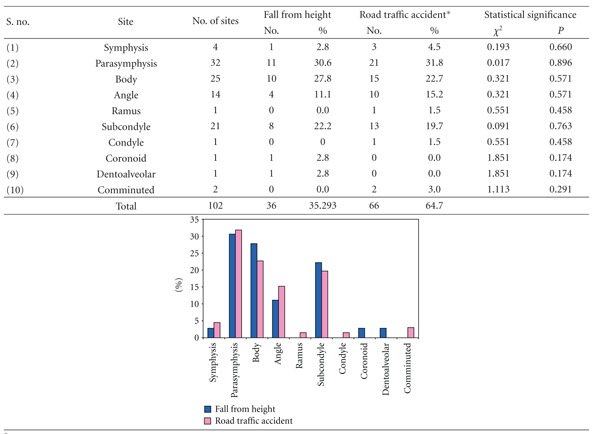

*Includes one case of hit against object.

**Table 9:** Age group versus number of fracture sites in mandible.